# Quarantine

**Published:** 1855-08

**Authors:** 


*Quarantine.*—Our New Orleans friends, after living a century or so in mud and Mississippi water, have finally decided that New Orleans is, *per se*, a very healthy city, and that such would be its reputation were it not for the proclivity manifested by the diseases of other cities and countries to fasten themselves upon it. Diseases are great travelers, and have their summer resorts as well as our fashionables. Just as the "upper ten" go to Saratoga and Niagara in the hot weather, so do yellow-fever and cholera go to New Orleans. It is a favorite stamping-ground for Blue Dick and Yellow Jack. Not that any Blue Dicks or Yellow Jacks ever originated in New Orleans. Oh no! they are always imported—visiting the place for reasons of their own, entirely incomprehensible. Occasionally they make a call on some other gulf town, but manifest a decided preference for New Orleans. No doubt Blue Dick and Yellow Jack have their reasons for their preference, but it would be unkind to inquire too closely into personal matters, and then, too, it might injure the immediate reputation of New Orleans were it to be understood that these lively friends of the undertaker were natives of, and active fellows in that city, carrying on a long established business with unparalleled enterprize and success.

But as some years ago the Vicksburg people expelled the gamblers, so now the New Orleans Board of Health have decided on keeping out the yellow fever and cholera by quarantine. It seems to be settled that the colored gentlemen alluded to always reach New Orleans by way of the Balize. In order to stop them here, it is only necessary to detain all vessels arriving from ports within the tropics for the brief period of twenty days.

The sea captain, who, after a long voyage from Rio Janeiro, arrives at the Balize, should rest satisfied there awhile. If he has not the yellow fever on board stowed away between decks "in the original package," we presume a three weeks' stay in the cheerful and salubrious region at the debouchure of the Mississippi, would conduce to his health in other respects, and give him plenty of time for moral reflection. The steamer from Chagres or San Juan, with its five or six hundred sea-sick passengers, can have plenty of time to blow out its boilers, and no doubt the exhausted passengers aforesaid would recruit rapidly under the sun of the Balize, and the fresh breeze from its bayous.

It is evident, then, that to put a stop to epidemics in New Orleans, it is only necessary to discontinue its commerce. For our part, we have no doubt the plan would succeed, and all the theories of Dr. Barton and others as to the domestic origin of yellow fever, would fall to the ground for want, of food to nourish them. The commerce being gone, the population follows, and by this short hand method the yellow fever is nowhere, and New Orleans becomes healthy by the removal of its inhabitants.

Whether this plan—inevitably successful as it must be—will be preferred to that which entails the expense of pavements, sewers and cleanliness upon the city, is no longer doubtful. We look upon the spectacle with the same intense admiration as we once felt for a gentleman, full of expedients, who avoided the cholera by blowing out his brains with a horse-pistol.

We notice, however, that Dr. Barton is in the opposition, and, in order that our article may not be too one-sided and illiberal, we append his letter of resignation as a member of the Board of Health and Quarantine.

*To the Honorable the Board of Aldermen of the City of New Or leans.*

Gentlemen: The undersigned respectfully returns to your honorable body the appointment you did him the honor to assign him as member of the Board of Health and Quarantine.

A sincere and ardent promoter of both for many years—yet he deems his usefulness to the city curtailed by the adoption—by a majority of the Board —of views he deems unnecessarily harsh and severe—not called for by existing circumstances, not corresponding with the enlightened age in which we live, and with no compensation for the injury it inflicts—he is not willing to be responsible for. This is more fully set forth in the protest annexed and prayed to be made part of this communication. It is signed by three members of the Board. Its record on the minutes has been refused by a majority of the Board, on a technical quibble as to time, which the undersigned trusts will not be followed by your honorable body.

The opinions of this community differ materially in relation to the efficacy of a Quarantine at all, in preventing the advent of yellow fever, but more, in relation to the mode of carrying it out. By enforcing it in such a way as to make it solely protective against a real, not a fictitious danger, by applying the remedy to the actual difficulty, to make only those coming from known sickly ports, or with sick or foul vessels, amenable for their own real condition, and that of their passengers and crew, or even where probable grounds of danger exists, and not encumber the commerce of the port (already much crippled) with restrictions which can effect no possible good to any one, are, in the opinion of the undersigned, the only rational grounds of Quarantine, and in which he regrets he has the misfortune to differ from a majority of his colleagues, and he respectfully asks to be relieved from responsibility for a course he cannot conscientiously approve.

Your obedient servant,

New Orleans, June 18, 1823. E. H. Barton.

